# Individual and community-level determinants of non-use of contraceptive among women with no fertility desire in Ethiopia: a multilevel mixed-effect analysis

**DOI:** 10.1186/s12982-022-00112-z

**Published:** 2022-04-02

**Authors:** Kusse Urmale Mare, Setognal Birara Aychiluhm, Abay Woday Tadesse, Osman Ahmed Mohammed

**Affiliations:** 1 Department of Nursing, College of Medicine and Health Sciences, Samara University, Samara, Ethiopia; 2 Department of Public Health, College of Medicine and Health Sciences, Samara University, Samara, Ethiopia

**Keywords:** Ethiopia, Fertility desire, Multilevel analysis, Non-use of contraceptives, Reproductive-age women

## Abstract

**Background:**

In Ethiopia, about two-third of women in the reproductive-age do not use any method of contraception. Moreover, evidence on non-use of contraceptives among women who do not have future fertility desires are limited. Therefore, this study intended to identify both individual and community-level determinants of non-use of contraceptives among this group in Ethiopia using a multilevel mixed effect analysis.

**Methods:**

Data retrieved from the demographic and health survey program official database website (http://dhsprogram.com) were used in this study. The suvey was conducting using a multistage cluster sampling technique and a weighted sample of 4398 reproductive-age women with no fertility desire was used in this study. Four models were fitted using a multilevel multivariable logistic regression to identify determinants of non-use of contraceptives and model with the lowest Akaike’s Information Criterion was selected as a best fitted model. Adjusted odds ratio with its corresponding 95% confidence interval was used to declare the statistical significance of the independent variables.

**Results:**

Overall, 65.3% [95% CI (63.9%, 66.7%)] of women with no fertility desire were not using any contraceptive method. Living in large central [AOR (95% CI)  0.45 (0.31, 0.67)] and metropolitan regions [AOR (95% CI) 0.39 (0.22, 0.68)] and being from household with middle [AOR (95% CI)  0.65 (0.42, 0.93)] and rich wealth index [AOR (95% CI)  0.67 (0.44, 0.98)] were negatively associated with non-use of contraceptives. Besides, being from a community with high women illiteracy [AOR (95% CI)  1.38 (1.15, 1.67)], being Muslim [AOR (95% CI)  1.86 (1.22, 2.85)], having history of pregnancy termination [AOR (95% CI) 1.59 (1.10, 2.31)], having a husband who desire to have more children [AOR (95% CI)  1.46 (1.02, 2.09)] were the positive determinants of non-utilization of contraceptives.

**Conclusion:**

Nearly two-third of reproductive-age women with no fertility desire in Ethiopia do not use any contraceptive method. Awareness creation interventions on the benefits of contraceptives targeting Muslim religion followers and improving women education and their economic empowerment at household level may decrease the proportion of non-use of contraceptives at a national level.

## Background

Contraception is one of the cost-effective safe motherhood initiatives implemented to improve maternal and child health through the promotion of optimal birth spacing [1, 2]. Existing evidence showed that the growing use of contraceptive methods is associated with reduction in fertility rates, unintended and high-risk pregnancies, and maternal and infant mortality [3–6]. Studies also revealed that contraceptive use is attributable to a decline in the number of unsafe abortions [3–5].

Despite their benefit in improving maternal and child health, a great proportion of women do not use contraceptives with a considerable variation within and across different geographical areas. Globally, less than half (43%) of married reproductive-age women who wanted to avoid pregnancy did not use any method of contraception in 2019, indicating a slow decline from 45% in 2000 [2, 6, 7]. In Sub-Saharan African countries, where the unmet need for family planning is high, nearly three-fourth (72%) of women of reproductive age do not use any contraceptive method, which ranges from 48% in Namibia to 96% in South Sudan [2, 6].

Studies in various regions of the globe show a disparity in the proportion of non-users of contraceptives among women. For instance, 87% of women in Burkina Faso, 2017 [8], 70% in Gambia, 2020 [9], 66% in Tanzania, 2020 [10], and 63% in Ghana, 2020 [11] do not use contraceptive methods. In Ethiopia, according to the 2016 demographic and health survey (DHS) report, 64% women do not use any method of contraception [12]. Furthermore, community-based cross-sectional studies in different parts of the country reported that non-utilization of contraceptives varied from 17 to 81% [13–20].

Evidences have shown that factors like women’s age [13, 18, 20–23], women education [13, 22–24], women occupation [18, 21, 22], husband education [15, 18, 21], place of residence [13, 16, 18, 25], number of children [13, 18, 22, 23], couple discussion on contraception [19, 21, 26], contraceptive knowledge [15, 20], distance to health facility [8, 27], wealth index [8, 13, 18, 22, 24], religion [13, 23, 24] and media exposure [13, 18] were found to predict whether a woman use or not use contraceptive methods.

In Ethiopia, different strategies were implemented to increase the uptake of contraceptive methods in the last decade. Implementation of the health extension programs to change attitude and improve awareness of the community about contraception was among the efforts taken to increase utilization of contraceptives [28, 29]. An increase in health workforce and expansion of health centers and health posts and upgrading of primary health care units to improve access to health care service were also the measures taken to partly remove health system barriers to contraceptive use [28]. Despite the efforts made at a national level, the proportion of women using contraceptive methods is low [12].

In Ethiopia, although different studies have been conducted to determine the predictors of non-use of contraceptives among women, some of them were limited to the specific geographical areas and used relatively a small sample size [14, 17, 20, 25, 26, 30–32]. In addition, these studies did not consider some community-level factors like region, residence, cluster, women illiteracy status, women’s non-exposure to mass media status. Moreover, evidence on non-use of contraceptives and its determinants among women with no fertility desire at a national level is scarce. In this regard, consideration of these community-level variables and using relatively large sample size could help to make inferences at a national level. Therefore, the current study was aimed to assess non-utilization of contraceptives and its determinants at individual and community levels among women with no fertility desire in Ethiopia using multilevel logistic regression analysis.

## Methods

### Data source, study period, and design

Secondary data analysis was performed using the data retrieved from the DHS program's official database website (http://dhsprogram.com), which was collected from January 18 to June 27, 2016. The Ethiopian Demographic and Health Survey (EDHS) is a nationally representative survey conducted every five years in the nine regional states (Afar, Amhara, Benishangul-Gumuz, Gambela, Harari, Oromia, Somali, Southern Nations, Nationalities, and People’s Region, and Tigray), and two administrative cities (Addis Ababa and Dire-Dawa) of Ethiopia [12].

### Sampling procedures

The survey employed a stratified two-stage cluster sampling technique using the 2007 population and housing census as a sampling frame. Sampling strata were formed by dividing each of the regions included in the survey into urban and rural settings. In the first stage, 645 enumeration areas (EAs) were selected with probability proportional to the EA size and with independent selection in each sampling stratum. In the second stage, on average 28 households were systematically selected. A total weighted sample of 15,683 reproductive-age women was used in the survey. This study used 4398 married, fecund reproductive age women with no fertility desire and a total of 268 women who were pregnant during the study period were excluded from the analysis since the survey did not collect any information on contraceptive utilization for pregnant women. Besides, those declared infecund (menopausal) and sterilized (respondent or partner) were excluded from the final analysis. The detailed sampling procedure is available in the full EDHS 2016 report [12].

### Study variables

The outcome variable of this study was current non-use of contraceptives among women with no fertility desire. For the analysis purpose, this variable was dichotomized into “no = 1” (for women who reported not currently using any contraceptive method) and “yes = 0” (for women reported currently using any contraceptive method). Fertility desire was determined from the variable “fertility preference” in the 2016 EDHS dataset. Accordingly, women who responded “no more children” for this variable were considered as women with “no fertility desire”, while those with the response “want within 2 years”, “wants after 2 years and above”, “wants, unsure timing” were taken as those with “future fertility desire”.

The predictor variables were classified into individual and community-level variables. Individual-level variables were age of women, women’s and partners’ education and occupation, wealth index, religion, household head, family size, parity, number of living children, women’s health care decision autonomy, history of pregnancy termination, heard about contraceptives, previous contraceptive use, and husband’s desire for children. The wealth index was recategorized as poor = 0 (for households in the poorest and poorer categories), middle = 1, and rich = 2 (for those in the rich and richest categories).

Community-level variables were region, residence, community-level women’s illiteracy status, and women’s non-exposure to media status. The geographical region was categorized into three regions (small peripheral, larger central, or metropolitan) based on their geopolitical features [33]. Small peripheral include Afar, Somali, Benishangul, and Gambella regions. The larger central regions include Tigray, Amhara, Oromia, and Sothern Nations Nationalities and Peoples Region (SNNPRs), while Metropolitan include Harari region, Dire Dawa, and Addis Ababa administrative cites. Community women’s illiteracy and non-exposure to media status were generated by aggregating individual characteristics within the cluster. The generated variables were further categorized as low and high based on the national median values of these variables. We preferred the median value because the proportion of women’s illiteracy and non-exposure to media among women was not normally distributed.

### Data management and statistical analysis

In the EDHS, sample allocation to different regions as well as urban and rural settings was not proportional. Thus, sample weights were applied to estimate proportions and frequencies to adjust disproportionate sampling and non-response. A full clarification of the weighting procedure was explained in the 2016 EDHS report [12]. Data were checked for missing values and data cleaning, exploratory analysis, variable recoding, labeling, categorization, and re-categorization were performed prior to analysis. The analysis was done using Stata version 16 and the presence of multicollinearity among independent variables was checked through variance inflation factor (VIF) taking a cut-off value of 10. However, the VIF value for all predictors was less than 10, indicating that there was no multi-collinearity between variables.

Bivariable multilevel logistic regression analysis was carried out to check the association between each predictor and the outcome variable. Variables with a p-value less than 0.25 in this analysis and those found important in the literature were considered as candidates for multivariable logistic regression analysis. In addition, the absence of collinearity between the independent variables, and the clinical importance of the independent variables were considered to select the candidate predictors for a multivariable multilevel logistic regression analysis model. To account for the clustering effects of the data, a multivariable mixed-effect logistic regression analysis was applied to determine the effects of each predictor on women’s’ non-use of contraceptives.

Following the selection of candidate variables for multivariable mixed-effect analysis, four models containing the variables of interest were fitted and the best-fitted model was selected. Model I (null model), a model without independent variables to test random variability in the intercept and estimate the intra-class correlation coefficient (ICC), proportion change in variance (PCV), and median odds ratio (MOR). Model II, a model with only individual-level explanatory variables. Model III, a model with only community-level explanatory variables, and Model IV (full model), which simultaneously examined the effect of both individual and community-level predictors on the outcome variable.

Akaike’s information criterion (AIC) value was used for model selection and the model with the lowest AIC value was considered as a best-fitted model for the final analysis. A p-value less than 0.05 in the multivariable mixed-effect logistic regression analysis was used to declare the statistical significance of the explanatory variables. Adjusted odds ratio (AOR) with a 95% confidence interval in the multivariable analysis was used to identify variables that have a statistically significant effect on women’s non-use of contraceptives.

## Results

### Socio-demographic and reproductive characteristics

Out of 4389 women included in the study, 4099 (93.2%) were from the large central regions and 3639 (82.7%) lived in rural settings. Regarding community level illiteracy and non-exposure to media, 2713 (61.7%) and 2384 (54.2%) were from the community with high women’s illiteracy and high non-exposure status to mass media, respectively. In this study, more than two-third (67.9%) of women did not attend formal education, and a half (51.4%) of them were non-working.

Concerning the reproductive characteristics, 3014 (74.0%) women gave birth at least four times and 2775 (82.2%) were non-autonomous on health care decisions. Four hundred seventy (10.7%) women had a history of pregnancy termination and 2372 (53.9%) had ever used contraceptives (Table 1).

**Table 1:** Socio-demographic and reproductive characteristics of women with no fertility desire in Ethiopia, 2016 (n = 4398)

Variables	Category	Frequency	Proportion
Region	Small peripheral	112	2.6
	Large central	4099	93.2
	Metropolitan	187	4.2
Residence	Urban	759	17.3
	Rural	3639	82.7
Women’s illiteracy status	Low illiteracy	1685	38.3
	High illiteracy	2713	61.7
Non-exposure to media status	Low non-exposure	2014	45.8
	High non-exposure	2384	54.2
Women of age	15–24	483	11.0
	25–34	1376	31.3
	35–49	2539	57.7
Religion	Orthodox	1956	44.5
	Protestant	1074	24.4
	Muslim	1288	29.3
	Others^a^	80	1.8
Women’s education	No schooling	2985	67.9
	Primary	1103	25.1
	Secondary and higher	310	7.0
Women’s occupation	Non-working	2136	48.6
	Working	2263	51.4
Partners’ education	No schooling	1739	51.5
	Primary	1258	37.3
	Secondary and higher	379	11.2
Household head	Male	3260	74.1
	Female	1138	25.9
Household wealth	Poor	1652	37.5
	Middle	865	19.7
	Rich	1881	42.8
Women’s health care decision autonomy	Non-autonomous	2775	82.2
	Autonomous	601	17.8
Family size	≤ 4	1080	24.6
	5–8	2682	61.0
	≥ 9	636	14.4
Parity	1–3	1069	26.0
	≥ 4	3041	74.0
Number of living children	0	302	6.9
	1–4	1866	42.4
	≥ 5	2230	50.7
Ever terminated pregnancy	No	3928	89.3
	Yes	470	10.7
Heard about contraceptives	Yes	3517	80.0
	No	881	20.0
Ever used contraceptive	No	2026	46.1
	Yes	2372	53.9
Husband’s desire for children	Wants the same number of children as spouse	1288	38.2
	Wants more children than spouse	960	28.5
	Want fewer children than spouse	268	7.9
	Don’t know	860	25.47

^a^*Others* Catholic, traditional, and other EDHS categories

### Non-utilization of contraceptives

In this study, non-utilization of contraceptive methods among women with no fertility desire was 65.3% [95% CI (63.9%, 66.7%)]. Of women not using contraceptive, 2390 (83.2%) resided in rural settings and about sixty-four percent of them were from the community with high women’s illiteracy. Moreover, the proportion of non-utilization of contraceptives among women from households with a poor wealth index was 41.4% (Table 3).

### Reasons for on-utilization of contraceptives

In this study, thinking that contraceptives are fatalistic (23.9%), fear of side effect (12.8%), menses not resumed since delivery (11.6%), currently breastfeeding (9.6%), religious prohibition, and infrequent sex (6.2%) were the reasons reported by the respondents for non-utilization of contraceptives (Table 2).

**Table 2:** Reasons for no non-utilization of contraceptive methods among women with no fertility desire in Ethiopia, 2016 (n = 4398)

Reasons	Proportion
Thinking contraceptives are fatalistic	23.9
Fear of side effects/health concerns	12.8
Menses not resumed since delivery	11.6
Currently breastfeeding	9.6
Religious prohibition	6.4
Infrequent sex	6.2
Husband and others opposition	2.3
Health facility related reasons*^a^	1.7
Others^b^	6.4

^a^lack of access/health facility too far, and preferred method not available

^b^inconvenient to use, interferes with body process, cost too much, know no method, and know no source, and other EDHS category

### Model building and selection

From the four models fitted, Model IV, a model with both individual and community-level predictors have the smallest AIC value. Hence, this model best fits the data. The random effect results were estimated using ICC, PCV, and MOR. The result of the random effects model showed that the variance of the random factor in the null model was 0.87 [95% CI 0.62, 1.20], indicating the existence of variation in the non-utilization of contraceptives across communities. Thus, to account for this variation, a multilevel logistic regression model was considered for further analysis.

The result of the random effect model showed that the intraclass correlation coefficient (ICC) was 21%, indicating that the correlation between women in the same EA on non-utilization of contraceptive was 0.21 i.e. 21% of the variation in the non-utilization of contraceptives among women with no fertility desire in Ethiopia could be attributed to EA difference. In the final model, PCV indicated that 32% of the variation in non-utilization of contraceptives across communities was explained by individual and community level predictors simultaneously. Besides, MOR indicated the unexplained community variation in non-utilization of contraceptives reduced from 2.41(null model) to 1.99 (full model). This shows that when all predictors are considered, the effect of clustering is still statistically significant (Table 3).

**Table 3:** Community-level variance and model comparison of two-level mixed-effect logistic regression model predicting non-utilization of contraceptive method among women with no fertility desire in Ethiopia, 2016

Random effect	Null model	Full model
Community-level variance	0.87	0.59
ICC (%)	21	15
PCV (%)	Reference	32
Median odds ratio (MOR)	2.41	1.99
Model fitness statistics (AIC)	5332	3964
Log likelihood	− 2670	− 1961

### Individual and community-level determinants of non-utilization of contraceptives

The multilevel multivariable mixed-effect logistic regression analysis showed that region and women’s illiteracy were the community-level variables significantly associated with the non-utilization of contraceptive methods. This analysis also found religion, wealth index, history of pregnancy termination, and husband’s desire for children as the individual-level determinants of women’s non-utilization of any contraceptive methods.

After adjusting for other covariates, the odds not using contraceptives among women in large central [AOR (95% CI) 0.45 (0.31, 0.67)] and metropolitan [AOR (95% CI)  0.39 (0.22, 0.68)] regions were reduced by 55 and 61%, respectively compared to those lived in small peripheral regions. Women's illiteracy status at the community level was positively associated with the non-utilization of contraceptives. For instance, women from the community with high women’s illiteracy status [AOR (95% CI)  1.38 (1.15, 1.67)] had a higher likelihood of being non-users of contraceptives compared to their reference group.

In addition, the odds of not using contraceptives among Muslim religion fellow were 86% higher compared to Orthodox religion followers [AOR (95% CI)  1.86 (1.22, 2.85)]. Likewise, wealth index was positively associated with non-utilization of contraceptive, where women in the households with middle [AOR (95% CI)  0.65 (0.42, 0.93)] and rich [AOR (95% CI)  0.67 (0.44, 0.97)] wealth status had lesser odds of being the non-users of contraceptives compared to those from poor households.

Non-use of contraceptives among women whose husbands desire to have more children [AOR (95% CI) = 1.46 (1.02, 2.09)] was increased by 46% compared to those women whose husbands have the same child desire with them. Similarly, the likelihood of not using contraceptives among women with a previous history of pregnancy termination [AOR (95% CI) 1.59 (1.10, 2.31)] were 55% higher compared to their counter group (Table 4).

**Table 4:** Multilevel multivariable logistic regression of the individual and community-related variables predicting non-utilization of contraceptive method among women with no fertility desire in Ethiopia, 2016 (n = 4398)

Predictor	Contraceptives use		Model II AOR (95%)	Model III AOR (95%)	Model IV AOR (95%)
	No	Yes			
Region					
Small peripheral	92 (3.2)	20 (1.3)		1	1
Large central	2664 (92.7)	1434 (94.1)	–	0.39 (0.27, 0.56)*	0.45 (0.31, 0.67)*
Metropolitan	118 (4.1)	70 (4.6)		0.40 (0.25, 0.64)*	0.39 (0.22, 0.68)*
Residence					
Urban	484 (16.8)	275 (18.1)	–	1	–
Rural	2390 (83.2)	1249 (81.9)		0.89 (0.58, 1.36)	
Community-level women’s illiteracy status					
Low illiteracy	1045 (36.4)	640 (42.0)	–	1	1
High illiteracy	1829 (63.6)	884 (58.0)		1.53 (1.16, 1.78)*	1.38 (1.15, 1.67)*
Community-level non-exposure to media status					
Low non-exposure	1290 (44.9)	724 (47.5)	–	1	–
High non-exposure	154 (55.1)	801 (52.5)		1.14 (0.85, 1.53)	
Religion					
Orthodox	1226 (42.6)	731 (47.9)	1		1
Protestant	611 (21.3)	463 (30.4)	0.91 (0.59, 1.41)	–	0.85 (0.55, 1.35)
Muslim	978 (34.0)	310 (20.4)	2.01 (1.34, 3.01)*		1.86 (1.22, 2.85)*
Others	60 (2.1)	20 (1.3)	3.10 (0.90, 6.24)		2.88 (0.83, 4.99)
Women’s education					
No schooling	1951 (67.9)	1034 (67.8)	1		
Primary	704 (24.5)	399 (26.2)	0.98 (0.71, 1.35)	–	–
Secondary and higher	219 (7.6)	91 (6.0)	1.06 (0.57, 1.96)		
Women’s occupation					
Non-working	1426 (49.6)	710 (46.6)	1	–	–
Working	1448 (50.4)	814 (53.4)	0.76 (0.57, 1.03)		
Household head					
Male	1952 (67.9)	1308 (85.8)	1	–	–
Female	922 (32.1)	216 (14.2)	1.54 (0.93, 2.57)		
Household wealth					
Poor	1190 (41.4)	462 (30.3)	1		1
Middle	526 (18.3)	339 (22.2)	0.63 (0.41, 0.96)*	–	0.65 (0.42, 0.93)*
Rich	1158 (40.3)	723 (47.4)	0.59 (0.40, 0.88)*		0.67 (0.44, 0.97)*
Family size					
≤ 4	775 (29.9)	305 (20.0)	1		
5–8	1614 (56.2)	1068 (70.1)	0.70 (0.44, 1.12)	–	–
≥ 9	484 (16.9)	151 (9.9)	1.51 (0.87, 2.61)		
Parity					
≤ 3	635 (24.4)	434 (28.8)	1	–	1
≥ 4	1968 (75.6)	1703 (71.2)	1.55 (1.18, 2.35)*		1.49(0.98, 2.27)
Heard about contraceptives					
No	2324 (80.9)	1193 (78.3)	1	–	–
Yes	550 (19.1)	331 (24.7)	0.98 (0.68, 1.40)		
Ever terminated pregnancy					
No	2546 (88.6)	1382 (90.7)	1		1
Yes	328 (11.4)	142 (9.3)	1.55 (1.07, 2.25)*	–	1.59(1.10, 2.31)*
Husband’s desire for children					
Wants the same number of -children as spouse	658 (33.6)	630 (44.4)	1		1
Wants more children than spouse				–	
Want fewer children than spouse	604 (30.8)	357 (25.1)	1.48 (1.03, 2.11)*		1.46(1.02, 2.09)*
Don’t know	162 (8.3)	105 (7.4)	1.38 (0.87, 2.0)		1.37(0.86, 2.19)

^*^Statistically significant at p-value < 0.05 in multivariable logistic regression analysis

## Discussion

This study revealed that 65.3% [95% CI (63.9%, 66.7%)] of married reproductive age women with no fertility desire do not use contraceptives, consistent with the findings from the previous studies in Ethiopia (65.1%) [13], Northern Ethiopia (64.6%) [14], Tanzania (65.7%) [10], India (63.3%) [23], and Ghana (63.1%) [11].

However, this finding was lower than the studies done in Cameroon, 87% [21] and different parts of Ethiopia [15–18, 22, 34], which reported non-utilization of contraceptives ranging from 70.8%-80%. Furthermore, this finding was also lower than a study in Gambia (69.6%) [35], Northwest Ethiopia (81.4%) [36], Nigeria (88.6%) [37], and Burkina Faso (87.3%) [8]. On the contrary, our finding was slightly higher than the studies in Southern (62.6%) [30], Central (62.1%) [38], and Northwestern (58.7%) [19] parts of Ethiopia. Besides, this findings was considerably higher than the result of the studies in Southwestern (17.4%) [17], and Southern (36.6%) [20] Ethiopia, Bangladesh (37.6%) [39], Zambia (40%) [40], India (46.4%) [41], and Qatar (52.2%) [42]. These discrepancies might be due to differences in the accessibility and availability of contraceptives and health facilities in the study areas. Differences in the design used, study population, the definition of the outcome variable, and characteristics of participants might be the possible explanation for the observed variations. For instance, most of the previous studies included all reproductive age women and limited to modern contraceptive methods. But the current study included both traditional and modern methods and the study population were women with no fertility desire.

The multilevel multivariable mixed-effect logistic regression analysis found that non-use of contraceptives was significantly associated with different community and individual-level factors. Regional variations were attributed to a significant difference in the non-use of contraceptives. For instance, compared to women in small peripheral regions, those in large central and metropolitan regions had a 55% and 61% decreased likelihood of being the non-users of contraceptives, respectively. This finding is supported by the results of the previous studies in Ethiopia [13, 18, 22] and Uganda [27], which reported a considerable variation in the utilization of contraceptives across different regions. Differences in the health system infrastructures, access to contraceptive services, and population characteristics might contribute to this variation.

Women from the community with high women’s illiteracy were 1.38 times more likely to be the non-users of contraceptives compared to their counter group. Similar associations were reported in the studies done in different parts of Ethiopia [14–16, 20, 22, 38, 42–44]. This could be explained by the fact that illiterate women might not have adequate knowledge about the potential consequences of unwanted pregnancy and high-risk fertility behaviors and thus may not use contraceptive methods. The negative effect of illiteracy on healthcare-seeking behavior might also contribute to a higher likelihood of non-use of contraceptives among women living in the community with high women illiteracy.

Consistent with the previous studies in Ethiopia [26, 38, 43], this study revealed that the odds of not using contraceptives for Muslim religion fellow were increased by 86% compared to Orthodox religion followers. This finding is also in agreement with the studies in India [23], Nigeria [45], and Tanzania [24], which found lower utilization of contraceptives among Muslim women. The negative influence of religious and cultural norms on the use and acceptance of contraceptive services [46] might have contributed to the variation in non-use of contraceptives among women with different religious background.

A significant reduction in the likelihood of being a non-user of contraceptives was observed as the household’s wealth status improves. Accordingly, compared to women from poor households, the odds of not using contraceptives were decreased by 35% for women in the households with middle wealth index and 33% for those from rich households. This finding is similar to the result of the studies in Ethiopia [13, 18, 22, 43] and Tanzania [24], which reported wealth status as an enabling factor for contraceptive utilization. This could be explained by the fact that women from the household with middle and rich wealth are less likely to have a problem in accessing contraceptives in terms of transport and service fee and thus less likely to be the non-users of contraceptives.

In this study, husbands' desire for children was also found to have a significant influence on women’s non-use of contraceptives. Women whose husbands desire to have more children had 1.46 times higher odds of being non-users compared to those whose husbands had the same child desire with them. This finding is supported by the result of a study in Ethiopia [15], and Zambia [47]. This might be due to the negative effect of gender inequalities [48] and the husband’s decision on women’s intention to use contraceptives [49] accompanied by the spousal desire for more children.

It was also revealed that the likelihood of not using contraceptives among women with a previous history of pregnancy termination was increased by 59% compared to those with no history of such obstetric event. This finding is in line with the previous study in Ethiopia which reported a decrease in the odds of contraceptive use among women who had ever terminated pregnancy [13, 25]. This could be because women with this history might have a desire to get pregnant again and thus might not use contraceptives.

Overall, our findings imply that qualitative studies are required to further discover socio-cultural and behavioral factors that hinder the utilization of contraceptive methods. In addition, systematic review and meta-analysis are recommended to know the pooled estimate and determinants of non-use of contraceptives at a national level to design better intervention strategies.

## Conclusion

Nearly two-third of fecund reproductive age women with no fertility desire in Ethiopia do not use any method of contraception. Region, community-level women's illiteracy, religion, household wealth, husband desire for children, and women’s history of pregnancy termination were the significant determinants for non-utilization of contraceptives. Awareness creation interventions on the benefits of contraceptives targeting Muslim religion followers and improving women's education and their economic empowerment at the household level may increase the uptake of the service and thus decrease the proportion of non-users.

## Strengths and limitations

The analysis of this study was based on a nationally representative large sample size and the most recent EDHS data, which was collected by standardized and validated data collection instruments. Besides, this study used an advanced statistical model, multilevel mixed effect modeling to account for the clustering effect of the EDHS data. However, due to the cross-sectional nature of the data, it is impossible to show the cause and effect relationship between the determinants and non-use of contraceptives. Recall bias may also be present because the study participants were asked about the events that took place five years or more preceding the survey.

## Data Availability

The raw dataset used and analyzed in this study can be accessed from the DHS website (http://www.measuredhs.com).

## Abbreviations

AIC: Akaike’s information criterion
AOR: Adjusted odds ratio
COR: Crude odds ratio
DHS: Demographic and health survey
EA: Enumeration area
EDHS: Ethiopian Demographic and Health Survey
ICC: Intra Class Correlation Coefficient
PCV: Proportional Change in Variance
